# Perfume Guns: Potential of Yeast Volatile Organic Compounds in the Biological Control of Mycotoxin-Producing Fungi

**DOI:** 10.3390/toxins15010045

**Published:** 2023-01-05

**Authors:** Safa Oufensou, Zahoor Ul Hassan, Virgilio Balmas, Samir Jaoua, Quirico Migheli

**Affiliations:** 1 Department of Agricultural Sciences, Desertification Research Centre (NRD), Università degli Studi di Sassari, Viale Italia 39, I-07100 Sassari, Italy; 2 Environmental Science Program, Department of Biological and Environmental Sciences, College of Arts and Sciences, Qatar University, Doha P.O. Box 2713, Qatar

**Keywords:** antagonistic microorganisms, biological control, microbial volatilome, mycotoxins, postharvest pathogens, toxigenic fungi, volatile organic compounds, yeast

## Abstract

Pathogenic fungi in the genera *Alternaria*, *Aspergillus*, *Botrytis*, *Fusarium*, *Geotrichum*, *Gloeosporium*, *Monilinia*, *Mucor*, *Penicillium*, and *Rhizopus* are the most common cause of pre- and postharvest diseases of fruit, vegetable, root and grain commodities. Some species are also able to produce mycotoxins, secondary metabolites having toxic effects on human and non-human animals upon ingestion of contaminated food and feed. Synthetic fungicides still represent the most common tool to control these pathogens. However, long-term application of fungicides has led to unacceptable pollution and may favour the selection of fungicide-resistant mutants. Microbial biocontrol agents may reduce the incidence of toxigenic fungi through a wide array of mechanisms, including competition for the ecological niche, antibiosis, mycoparasitism, and the induction of resistance in the host plant tissues. In recent years, the emission of volatile organic compounds (VOCs) has been proposed as a key mechanism of biocontrol. Their bioactivity and the absence of residues make the use of microbial VOCs a sustainable and effective alternative to synthetic fungicides in the management of postharvest pathogens, particularly in airtight environments. In this review, we will focus on the possibility of applying yeast VOCs in the biocontrol of mycotoxigenic fungi affecting stored food and feed.

## 1. Introduction

It is estimated that about one third of agricultural commodities produced worldwide are spoiled or wasted during the postharvest stages [1]. The reported figures range between 10–40%, but may reach 50–70% for some regions and crops where high relative humidity rates or unsuitable storage technologies are common [2,3,4,5,6]. Pathogenic fungi of the genera *Alternaria*, *Aspergillus*, *Botrytis*, *Fusarium*, *Geotrichum*, *Gloeosporium*, *Monilinia*, *Mucor*, *Penicillium*, and *Rhizopus* are considered to be those most frequently responsible for postharvest diseases in stored commodities, including fruits, vegetables, roots, pulses, and cereals. Some species of *Alternaria*, *Aspergillus*, *Fusarium* and *Penicillium* also produce mycotoxins, i.e., secondary metabolites contaminating food and feed [7,8]. Common mycotoxins include aflatoxins (AFs), ochratoxin A (OTA), deoxynivalenol (DON) and its acetylated forms 3 acetyl-deoxynivalenol (3-ADON) and 15-acetyl-deoxynivalenol (15-ADON), patulin (PAT), zearalenone (ZEN), and fumonisins (FUMs). The adverse effects of mycotoxins may comprise mild gastrointestinal disorders as well as chronic and acute syndromes, including immunosuppressive and carcinogenic effects, with severe consequences on human and non-human animals upon ingestion of contaminated food and feed [9,10].

The temperature, humidity, and CO_2_ concentration trends associated with climate change and their effects on fungus and plant physiology are expected to exacerbate the contamination of food and feed commodities with mycotoxins and masked mycotoxins, thereby posing unprecedented global challenges to food and feed safety standards worldwide [11,12].

The distribution of synthetic fungicides still represents the most common approach to control postharvest fungal pathogens, particularly in the case of fresh-fruit rots. Nonetheless, increasingly stringent regulatory policies have been enforced during recent years due to growing concerns over human health, pollution by chemical residues, and the selection of fungicide-resistant pathogens [13]. This has boosted the development of biological control as a valuable approach to cope with postharvest pathogens and several effective formulations based on microbial biocontrol agents (BCAs) are now commercially available [14,15,16,17].

Yeasts offer many advantages when designing effective BCAs; their yeast-like morphology allows straightforward culturability in fermentors, effective formulation, and multipurpose application options [15]. Their single-celled morphology favours adhesion and sometimes biofilm formation onto the fruit carposphere or on wound inner surfaces, allowing environmental persistence, competitiveness, and improved biocontrol effectiveness [18,19]. Moreover, yeasts have been traditionally used for thousands of years in the food and beverage industry, they are often consumed directly as food supplements, and are generally regarded as safe. For these reasons, the application of antagonistic yeasts in crops or food products provokes less concern in the public and in regulatory authorities compared with bacteria or filamentous fungi [20].

The biocontrol mechanisms employed by yeast BCAs are diverse, and may include competition for space and nutrients, mycoparasitism, induction of host resistance, or antibiosis [14,15,16,21,22,23]. Among the broad range of antimicrobial molecules, volatile organic compounds (VOCs) are now attracting the attention of scientists for the practical advantages of their potential application. VOCs are generally composed of a blend of volatile metabolites that may exert a strong inhibitory activity towards other organisms. Their potent bioactivity, along with the lack of reported significant effects on consumers’ health or the environment, have encouraged R&D efforts on VOCs as a sustainable replacement for synthetic fungicides in the control of postharvest pathogens, particularly in closed environments, or when the pathogen and its antagonistic microbe are not in direct contact [24,25].

Over 20 years ago, Strobel and co-workers [26] reported on the VOC-mediated biocontrol activity of a novel endophytic fungus from *Cinnamomum zeylanicum*. This serendipitous discovery opened an authentic Pandora’s box in the research on microbial VOCs and on their key role in biological control. The “marvellous” [27] *Muscodor albus* isolate 620, recently reclassified as *Induratia alba* [28], proved able to produce a wide range of volatile compounds, including alcohol-, acid-, ester and terpenoid derivatives with antimicrobial properties against many postharvest pathogens, including mycotoxigenic types [29]. Since then, many other microorganisms (filamentous fungi, yeasts and bacteria) have been reported to produce bioactive volatile metabolites, potentially applicable to agricultural commodities during storage.

Microbial VOCs are small (typically less than 300 Da, and up to 20 carbon atoms), odorous molecules, containing a lipophilic moiety, with low water solubility, high vapour pressure and low boiling point. VOCs encompass a wide range of molecular classes, including alcohols, aldehydes, benzene derivatives, cyclohexanes, heterocyclic compounds, hydrocarbons, ketones, phenols, thioalcohols and thioesters [30,31]. These compounds are generally effective at low concentration [32] and may diffuse in air and water, thereby representing ideal volatile messengers in distant interactions [33,34,35]. Besides exerting a direct inhibitory activity, microbial VOCs may play a key role in complex intra- and interspecific interactions, e.g., antibiotic resistance, cell-cell communication, sexual differentiation, morphogenesis [36,37], plant growth promotion [38,39,40,41], or the induction of plant resistance towards biotic and abiotic factors [42,43,44].

In most instances, several VOCs may be released by the same emitting organism, and the term “volatilome” was first proposed by Maffei et al. [45] to define these heterogeneous blends of metabolites. Even the same microbial strain may produce different VOCs depending on environmental factors, such as the age, the availability of nutrients, the growth temperature, the exposure to radiation, or the presence of other (micro)organisms [46,47,48,49,50].

Due to their physical nature, microbial VOCs are particularly promising as biofumigants under air-tight environments as they allow rapid saturation of the atmosphere with bioactive concentrations. The application of microbial VOCs has been reported as effective and technologically feasible in the biological control of many postharvest pathogens—including mycotoxin-producing fungi—on a wide range of stored commodities, such as fresh fruits, vegetables, nuts, grains, pulses and roots [25,30,51,52,53].

In this review, we will report the most relevant advancements on the use of yeast and yeast-derived VOCs as biofumigants in the control of mycotoxigenic fungi commonly found on food and feed commodities, with emphasis on the post-harvest stages.

## 2. Yeast Volatilome

Yeasts are able to release a panoply of volatile metabolites, which, besides contributing to determining their fragrance, may play a driving role in their ecological relationships. Yeast VOCs can be produced from both primary and secondary metabolism, and include alcohols, thioalcohols, thioesters, phenols, terpenes, hydrocarbons, heterocyclic compounds, aldehydes, and ketones [25,54]. Among the main yeast VOCs generated from the catabolism and fermentation of carbohydrates, ethanol is the most relevant, being produced both under anaerobic conditions via fermentative consumption of pyruvate, or aerobically at high glycolytic fluxes to re-oxidize NADH generated upon glucose oxidation. Acetaldehyde is the metabolic precursor of ethanol, and represents a leakage product of ethanol fermentation. Another fermentative byproduct is 2,3-butanediol, along with its precursor metabolite acetoin.

Other yeast VOCs (also known as “fusel alcohols”) are generated upon the Ehrlich catabolic pathway from amino acids such as isoleucine, leucine, methionine, phenylalanine, tryptophan, tyrosine, and valine. These compounds are generally considered troublesome when present at high concentration in fermented foods and beverages, as they contribute to ‘off’ flavours, but many of them were identified as bioactive against postharvest and toxigenic fungi. Among the most frequently detected compounds are 3-methyl-1-butanol (isoamyl alcohol) and 2-methyl-1-propanol (isobutanol), deriving from the degradation of leucine and valine; the aromatic 2-phenylethanol (end product of phenylalanine), which beside being used as rose-like fragrance in the perfume industry [55,56], bears powerful antimicrobial properties [57,58,59,60,61,62,63]; methional and dimethyldisulfide (derived from the catabolism of sulphur-containing amino acids, such as methionine and cysteine [54].

Several acetate esters (e.g., ethyl acetate, isoamyl acetate, isobutyl acetate, and phenyl ethyl acetate) derive from the condensation of carboxylic acids with acetyl coenzyme A and are often found in fermented beverages, where they confer various fruity aromas. These volatiles were also identified as inhibitors of fungal growth and mycotoxin biosynthesis on many toxigenic species (e.g., [64,65,66,67,68,69]).

Another source of volatile metabolites is represented by the synthesis and degradation of fatty acids, such as decanoic, octanoic, hexanoic, propanoic and butanoic acid. Upon decarboxylation, fatty acids may generate alkanes, 1-alkenes, and methyl-ketones, whereas their reduction leads to the release of the respective aldehydes and 1-alkanols. Long-chain alkanes (nonadecane, eicosane, docosane, heptacosane, hexatriacontane, and tetracosane) produced by *Kluyveromyces marxianus* were found able to reduce OTA biosynthesis potential of *Penicillium verrucosum* and *Aspergillus carbonarius* [70].

Lactones (e.g., γ-decalactone, γ-butyrolactone, 4-hydroxy cis-6-dodecenoic acid-γ-lactone) are generated by fatty acids via subsequent hydroxylation, β-oxidation and intramolecular esterification steps, ultimately leading to cyclization [54].

In addition to the previously mentioned acetate esters, fatty acid ethyl esters may be produced from volatile medium-chain-length fatty acids upon reaction with ethanol [71]. These include ethyl hexanoate and ethyl octanoate [71], having industrial interest due to their fruity or floral flavours. Some derived compounds (3-methylbutyl hexanoate, 3-methylbutylpentanoate, 2-methylpropyl hexanoate, and pentylhexanoate) were also found as major components of the *Candida sake* volatilome during the interaction with the patulin-producer *Penicillium expansum* [72].

Yeasts may also transform plant terpenes into their oxygenated derivatives, terpenoids, that have widespread applications as pharmaceuticals, flavours, fragrances, or fuel alternatives [73]. While yeasts have been traditionally considered as unable to achieve *de novo* synthesis of terpenes or terpenoids, with the exception of geraniol and the well-known quorum sensing molecule farnesol [74], new pathways are now being explored in metabolically engineered *Saccharomyces cerevisiae* [75,76,77].

## 3. VOCs-Mediated Control of Postharvest Mycotoxigenic Fungi

### 3.1. Alternaria

Species within the genus *Alternaria* are among the most common postharvest pathogens of fresh fruits and vegetables [78,79]. Several species are able to produce mycotoxins, such as tenuazonic acid, alternariol, alternariol methyl ether, altenuene, and altertoxins. *Alternaria alternata*, the most common species in stored commodities, is also considered the most important toxigenic species. *A. alternata* produces a wide array of toxic metabolites, including the benzopyrone derivatives alternariol, alternariol methyl ether, altenuene, tenuazonic acid (a tetramic acid derivative), altertoxin-I (a perylene derivative; [78]). Many other *Alternaria* species produce mycotoxins: for instance, *A. brassicae*, *A. capsici-annui*, *A. citri*, *A. cucumerina*, *A. dauci*, *A. kikuchiana*, *A. longipes*, *A. porri*, *A. solani*, *A. tenuissima*, and *A. tomato* produce alternariol and alternariol monomethyl ether, whereas *A. capsici-annui*, *A*. *citri*, *A. japonica*, *A. kikuchiana*, *A. longipes*, *A. porri*, *A. radicina*, *A. tenuissima* and *A. tomato* are able to produce tenuazonic acid (reviewed by Barkai-Golan [78]).

Tenuazonic acid is toxic to several animal species, while alternariol may affect DNA integrity in human colon carcinoma cells [80,81]. Altertoxin II is mutagenic in cultured Chinese hamster V79 cells, and reported to be at least 50 times more potent as a mutagen on mammalian cells than the common *Alternaria* toxins, alternariol and alternariol methyl ether [82]. *Alternaria* mycotoxins are frequently detected in fresh fruit and vegetables (apples and derived products, Japanese pears, prune nectar, mandarins, melons, olives, raspberries, red currant, carrots, pepper, red pepper, tomatoes, and tomato products), in oilseed rape meal, sunflower seeds, sorghum, wheat, barley, oats and edible oils [78,79,80].

Despite considerable efforts in the use of antagonistic yeast to control postharvest decay of *Alternaria* spp. (e.g., [83,84,85,86], among others), relatively few reports are available on the efficacy of yeast VOCs as inhibitors of these pathogens (Table 1).

The first report of the inhibitory activity of yeast VOCs against *Alternaria* spp. appeared in 2017 [72]: a collection of yeast isolates from Antarctica was explored in the search for potential biocontrol agents among psychrotrophic, non-pectinolytic strains able to grow in apple juice. Two isolates of *Candida* sake were selected for their ability to reduce apple rot caused by *P. expansum* and *B. cinerea* on apples stored at 0–1 °C. The *C. sake* volatilome, analysed by GC-MS, was composed of 11 VOCs, the most abundant of which being 3-methylbutyl hexanoate, 3-methylbutylpentanoate, 2-methylpropyl hexanoate, and pentylhexanoate. The inhibitory activity of yeast VOCs was confirmed on different postharvest pathogens, including *A. alternata*, *A. tenuissima*, and *A. arborescens*, growing *in vitro* on apple juice agar [72].

Oro et al. [67] analysed the major volatiles released by antagonistic *Wickerhamomyces anomalus*, *Metschnikowia pulcherrima* and *S. cerevisiae* and found differing amounts of ethyl acetate, isoamyl acetate, ethyl butyrate, ethyl hexanoate, phenylethyl acetate, 2-phenylethanol, and isobutanol in their volatile blends. In a dual plate test, *W. anomalus* volatiles inhibited *A. alternata* vegetative growth by 47%. Since ethyl acetate was the mainly represented VOC of *W. anomalus*, this compound was tested alone, and at the concentration of 0.718 mg/cm^3^, it significantly inhibited postharvest decay on strawberry, mainly caused by *Botrytis cinerea* [67].

Yalage Don et al. [63] designed a new setup for automated extraction of VOCs by headspace-solid-phase microextraction-gas chromatography-mass spectrometry (SPME-GC-MS). The system allowed quantification of volatiles during the interaction between *Aureobasidium pullulans* and *A. alternata* (in addition to *B. cinerea*). By this approach, the authors identified 14 VOCs, including seven alcohols, three ketones, and four esters. Ethanol and 2-phenylethanol were identified as the key inhibitory compounds in the biocontrol efficacy of *A. pullulans* against the pathogens. By using a response surface modelling approach, the authors also designed an optimised cocktail of ethanol, 3-methyl-1-butanol, 2-methyl-1-propanol and 2-phenylethanol to inhibit *in vitro* vegetative growth of both *A. alternata* and *B. cinerea* [63]. The mechanism of action of this volatile blend was investigated in depth in a subsequent study by Yalage Don et al. [112]. Based on previous evidence of the possible relationship between bioactive VOCs of *S. cerevisiae* and the accumulation of reactive oxygen species (ROS) leading to oxidative stress and lipid peroxidation in *Guignardia citricarpa* [113], *Colletotrichum gloeosporioides* and *Colletotrichum acutatum* [114]), Yalage Don et al. [112] demonstrated that the optimised *A. pullulans* VOCs cocktail (ethanol, 2-methyl-1-propanol, 3-methyl-1-butanol, and 2-phenylethanol) also induces ROS accumulation and electrolyte leakage in *A. alternata*. Additionally, altered cell wall structures were evidenced by scanning electron microscopy upon exposure to *A. pullulans* VOCs [112].

### 3.2. Aspergillus

The genus *Aspergillus* includes about 340 known species; many of them are able to produce a broad range of mycotoxins [115,116] that are harmful to humans and animals upon ingestion. Some pathogenic *Aspergillus* spp. can infect plants and plant commodities before and/or after harvest, often acting as opportunistic storage moulds. As a consequence of infection, contamination of foods and feeds by mycotoxins occurs, with aflatoxins, OTA, and, to a lesser extent, fumonisins being the most relevant toxic metabolites.

Aflatoxins B1, B2, G1, G2, mainly produced by aflatoxigenic strains of *A. flavus* and *A. parasiticus*, are frequently found on peanuts, fresh fruit (e.g., date, apple, orange), dried fruit (figs, raisin), and nuts (pistachio, hazelnut, almond). Aflatoxin B1 is by far the most toxic compound, due to its extreme hepatotoxicity and hepatocarcinogenicity. AFB1 is considered as the most potent naturally occurring carcinogen [117], and also the most common product of toxigenic strains. The International Agency for Research on Cancer (IARC) has assigned all aflatoxins to group 1 (“carcinogenic to humans”; [117]).

Ochratoxin A (OTA) is produced by several species in the closely related sections *Circumdati*, *Flavi* and *Nigri*—the latter only in subgenus *Circumdati* [116], including *A. ochraceus*, *A. carbonarius*, *A. niger*, *A. verrucosum*, *A. steynii*, *A. westerdijkiae*, as well as *A. elegans*, *A. sclerotiorum*, *A. sulphureus*, *A. tubingensis*, *A. aculeatus*, *A. alliaceus*, *A. melleus*, and others [118]. Ochratoxins are frequent contaminants in cereals, coffee, cocoa, grape-derived foods and beverages, spices, and many other agricultural commodities. OTA is a potent nephrotoxin, presenting carcinogenic, teratogenic, and immunotoxic activity in rats and, possibly, in humans. This has led to the classification of OTA in IARC group 2B (“sufficient evidence of carcinogenicity in experimental animals”). However, new insights on the genotoxicity of OTA, its role in oxidative stress, and the identification of epigenetic factors involved in OTA carcinogenesis may soon provide strong evidence that OTA carcinogenicity is also mediated by mechanisms operating in humans. Under such circumstances, it would not be inappropriate to consider an upgrade of its classification from Group 2B to Group 2A (“probably carcinogenic to humans” [119]).

The carcinogenic mycotoxins fumonisin B2 and fumonisin B4, which had been previously considered specific metabolites of *Fusarium* spp., were detected for the first time in the industrially important *Aspergillus niger* [120,121]. Fumonisins are hepatotoxic and nephrotoxic, with potential carcinogenic effects on rats and mice. These mycotoxins cause lung oedema in pigs, leukoencephalomalacia in horses, hepatocarcinoma in laboratory animals and, most importantly, esophageal cancer in humans [119]; therefore, they have been assigned to Group 2B by IARC (“possibly carcinogenic to humans” [117]). Noteworthily, *A. niger* is one of the most common contaminants of plant-derived products, and certain strains were shown to produce both OTA and fumonisins. Therefore, some commodities may potentially be contaminated with two types of carcinogenic mycotoxins from the same species [122].

Many other toxic metabolites are produced by *Aspergillus* spp., the most important of these being sterigmatocystin (produced by *A. flavus*, *A. flavipes*, *A. nidulans* and *A. versicolor*), cyclopiazonic acid (*A. flavus*, *A. tamarii* and *A. versicolor*), aflatrem (*A. flavus*), citrinin (*A. flavipes*, *A. carneus*, *A. niveus* and *A. terreus*), gliotoxin (a virulence factor in the human pathogenic species *A. fumigatus*), and patulin (*A. terreus* [115]).

The inhibitory effects of VOCs produced during wet processing of Arabica coffee in Tanzania by predominant yeast species *Pichia anomala*, *P. kluyveri* and *Hanseniaspora uvarum* were tested for the first time by Masoud et al. [64] against *A. ochraceus*. Yeast VOCs inhibited fungal growth and OTA production, with the two *Pichia* spp. displaying the strongest efficacy. The main esters and alcohols produced by the three yeasts were ethyl acetate, isobutyl acetate, 2-phenyl ethyl acetate, ethyl propionate and isoamyl alcohol. The individual compounds also affected fungal growth, with 2-phenyl ethyl acetate being the most effective inhibitor, leading to complete suppression of mycelial growth and OTA production at 48 μg/l headspace [64,65,66].

Surprisingly, no further evidence of the role of yeast VOCs as inhibitors of Aspergilli was published during the following decade, until Fiori et al. [87] reported the biocontrol efficacy of four low- or non-fermenting yeast (*Candida friedrichii*, *Candida intermedia*, *Cyberlindnera jadinii*, and *Lachancea thermotolerans*) against two isolates of *A. carbonarius*, and their ability to remove OTA from grape juice. This biological treatment was proposed to meet the requirements of Islamic dietary laws concerning the absence of residual alcohol in *halal* beverages. VOCs produced by *C. friedrichii* reduced *A. carbonarius* vegetative growth significantly, resulting in 33–58% reduction of colony diameter. *A. carbonarius* colonies exposed to yeast VOCs did not sporulate, and presented a white mycelium. However, when hyphal tips and mycelium fragments were transferred on fresh PDA and incubated at 25 °C, the typical black sporulating colonies developed, indicating that the anti-sporulating effect is reversible. None of the yeast-isolate VOCs was able to significantly reduce the incidence of infection by *A. carbonarius* on detached grape berries [87]. Aiming to further characterise the effect of VOCs produced by the same four biocontrol yeasts, Farbo et al. [61] reported that, in addition to inhibiting vegetative growth and sporulation, the volatile compounds reduced the production of OTA by *A. carbonarius* and *A. ochraceus*; OTA content released by *A. carbonarius* in the medium dropped from 7613–13,883 ng/g in the unexposed control to 0.1–135 ng/g upon exposure to yeast volatiles, whereas in *A. ochraceus* OTA release was reduced from 19,609–42,960 to 2.7–940 ng/g [61]. Upon exposure to yeast VOCs, gene expression was also affected in both fungi, as confirmed by downregulation of polyketide synthase, non-ribosomal peptide synthase, monooxygenase, and the regulatory genes *laeA* and *veA*. Headspace-solid-phase microextraction-gas chromatography-tandem mass spectrometry (HS-SPME-GC–MS) analysis identified 2-phenylethanol as the main yeast volatile [61].

A proteome analysis was later performed by the same group on *A. carbonarius* exposed to either *C. intermedia* volatilome or 2-phenylethanol, in order to understand whether the inhibitory effects of the yeast VOCs are solely attributable to 2-phenylethanol or if all volatile components are required to achieve an effective control of the fungal growth and metabolism. Yeast VOCs targeted several metabolic routes, including a drastic reduction in protein biosynthesis, proliferative activity, mitochondrial metabolism, and detoxification of toxic substances [62]. Exposure to 2-phenylethanol only partially mimicked the metabolic effects observed by the whole yeast volatilome, with protein biosynthesis and proliferative activity being reduced when compared with the control samples, but still less than in the VOC-exposed condition. This study represented the first proteome-based investigation on the effects of yeast-derived volatilome and 2-phenylethanol on the metabolism of a mycotoxigenic fungus [62].

In 2015, Nally and co-workers [88] tested a collection of viticultural yeasts (31 *Saccharomyces* and 28 non-*Saccharomyces*) for their ability to inhibit the vegetative growth of fungi isolated from sour and grey rot in grape. Among other mechanisms of action, representative strains of *S. cerevisiae*, *S. kluyveri*, *C. sake*, *Schwanniomyces vanrijiae* (syn. *Debaryomyces vanrijiae*), and *Wickerhamiella versatilis* (syn. *C. versatilis*) produced bioactive VOCs with inhibitory activity against different *Aspergillus* species (*A. carbonarius*, *A. caelatus*, *A. terreus*, and *A. versicolor* [88]).

Alasmar et al. [70] demonstrated the strong inhibitory activity of a Qatari isolate of *Kluyveromyces marxianus* towards the vegetative growth and mycotoxin biosynthesis potential of key toxigenic fungi, including *A. ochraeus*, *A. westerdijkiae*, *A. carbonarius*, *A. niger*, and *A. parasiticus*. *In vitro* radial growth inhibition ranged from 85% for *A. ochraeus* to 52% for *A. parasiticus* [70], while VOCs were able to reduce OTA biosynthesis potential of *A. carbonarius* by almost 99%. GC/MS-based analysis of yeast VOCs highlighted the presence of long-chain alkanes, including nonadecane, eicosane, docosane, heptacosane, hexatriacontane, and tetracosane. Finally, *in vivo* exposure to yeast VOCs protected grape berries from infection by *A. carbonarius*, as a consequence of complete inhibition of fungal spore germination, thereby suggesting a strong biopreservation potential [70].

A collection of 32 yeast isolates (including *Candida*, *Meyerozyma*, *Pichia*, *Wickerhamomyces*, *Rhodotorula* and *Saccharomyces* spp.) was screened by de Souza et al. [89] to isolate candidate biocontrol agents able to reduce OTA contamination during coffee post-harvest processing. *Saccharomyces* spp. produced VOCs with inhibitory activity on vegetative growth and sporulation of both *A. carbonarius* and *A. ochraceus* [89].

Similarly to what was reported by Farbo et al. [61], Hua et al. [57] used SPME-GC/MS analysis on VOCs released by the *P. anomala* biocontrol strain WRL-076, able to reduce AF contamination on tree nuts, and identified 2-phenylethanol as the major volatile component. This compound inhibited spore germination and AF production of *A. flavus in vitro*; AFB1 production decreased by 30, 35, and 96% at the 2-phenylethanol concentration of 0.2, 0.5, and 1.0 μL/mL, respectively, while at the concentration of 2 μL/mL of 2-PE, AFB1 was no longer detectable in fungal cultures [57]. Significant (>10,000-fold) downregulation of AF biosynthesis genes was evidenced by RT-PCR upon exposure to 2-phenylethanol, as well as altered gene expression patterns of several chromatin modifying genes, suggesting that this compound may be able to elicit epigenetic regulation of AF biosynthetic genes [57].

Using RNA-Seq technology, Chang et al. [59] were able to evaluate the temporal transcriptome response of *A. flavus* NRRL3357 to 2-phenylethanol applied at a sub-inhibitory level (i.e., 1 μL/mL). During a 72 h experiment, 131 out of 13,485 *A. flavus* genes were affected, with 82 genes (encoding conidiation proteins and involved in cyclopiazonic acid biosynthesis) downregulated. During the first 48 h treatment, the expression of all genes in the aflatoxin gene cluster were also significantly decreased. Gene Ontology (GO) analyses showed that exposure to low levels of 2-phenylethanol stimulated active growth of *A. flavus*, but decreased branched-chain amino acid degradation. Since secondary metabolism takes place after active growth has ceased, growth stimulation suppressed the expression of aflatoxin biosynthesis genes. The authors hypothesised that increased activities in degradation pathways for branched-chain amino acids may be required for the activation of the aflatoxin pathway [59].

The same four low- and non-fermenting yeasts previously characterised by Fiori et al. [87] and by Farbo et al. [61] were also tested against other mycotoxigenic fungi, including the AF producer *A. parasiticus* [90]. Besides inducing a significant reduction of vegetative growth, yeast VOCs suppressed AF biosynthesis *in vitro*, leading to up to 96% AF reduction in the case of a single-spore *A. parasiticus* colony. Recently, VOCs emitted by the strain of *C. jadinii* were shown to significantly reduce the colony radial growth of *A. parasiticus*, *A. niger* and *A. verrucosum*, grown *in vitro* [100]. In both reports, the authors hypothesised that 2-phenylethanol may play the key role as mycotoxin inhibitor.

Along with phenylethyl acetate, several esters, alcohols, terpenes, ketones, aldehydes, and aromatic hydrocarbons, 2-phenylethanol was also reported in the volatilome of *Issatchenkia orientalis* (syn. *Pichia kudriavzevii*), *Pichia occidentalis*, *Meyerozyma guilliermondii*, and *Meyerozyma caribbica*, responsible for the inhibition of mycelial *in vitro* growth of *A. flavus* and other fungi [69].

Moradi et al. [91] evaluated the biocontrol efficacy of 13 yeast isolates from soil and pistachio nuts sampled in Iranian pistachio orchards. In dual-culture tests, the vegetative growth of *A. flavus* was reduced by 13–31%, while aflatoxin B1 production was diminished by 90.6–98.3%. The effective isolates were identified as *I. orientalis* and *L. thermotolerans* [91].

Out of 366 epiphytic and endophytic yeast isolated from the leaves of rice, sugarcane, and corn grown in Thailand, 49 isolates produced VOCs; a strain of *Candida nivariensis* proved the most effective inhibitor of vegetative growth (65% inhibition) and spore germination (49% inhibition) of *A. flavus in vitro*. Moreover, the same isolate was able to reduce AF biosynthesis (75% inhibition) when growing in corn kernels. GC/MS analysis identified 1-pentanol as the major volatile component [92]. The same authors tested the ability of an isolate of *Kwoniella heveanensis* from corn leaves, whose VOCs were able to reduce AFB1 production in corn kernels by up to 96%. In this isolate, the volatilome was composed by a blend of volatiles, including 3-methyl-1-butanol, 2-methyl-1-butanol, hydrazine-1-1-dimethyl, and butanoic acid-3-methyl [93].

The inhibitory potential of VOCs released by two strains of *Hanseniaspora opuntiae* and *Hanseniaspora uvarum* on growth, on the expression of the regulatory gene *aflR*, and on AF mycotoxin production by *A. flavus*, was tested *in vitro* by Tejero et al. [95]. The two isolates produced different VOC blends (see Table 1), but both significantly inhibited the vegetative growth, mycotoxin biosynthetic gene expression, and aflatoxin (AFB1 and AFB2) production [95].

Recently, the same research group has tested three key components of the volatile blend produced by *H. opuntiae* and *H. uvarum* (octanoic acid, 2-phenylethyl acetate, and furfuryl acetate) on radial colony growth, spore germination, gene expression, and on the production of AFs and OTA by two representative strains of *A. flavus* and *A. niger* on dried fig-based agar. This substrate was selected since both fungi commonly colonise dried figs in pre- and postharvest, and contamination of fruit with AFs and OTA occurs frequently. The expression of two key AF and OTA biosynthetic genes (*aflR* and *pks*, respectively) was repressed in the two tested species upon exposure to yeast VOCs, albeit the repressing effect varied upon the exposure time. Two of the three volatiles, namely 2-phenylethyl acetate and furfuryl acetate, completely inhibited *A. flavus* growth in dried figs during 30 days of storage [97].

Soil yeast isolates collected from Western and Eastern Ghats of India were tested by Natarajan et al. [96] for their ability to reduce aflatoxin production by *A. flavus*. The most effective were identified as *S. cerevisiae*, *Suhomyces xylopsoci*, *I. orientalis*, and *C. tropicalis*. Volatiles of *S. cerevisiae* effectively suppressed the vegetative growth (up to 92.1%), inducing hyphal deformity and mycelial damage. Aflatoxin B1 production was almost completely (99%) suppressed in the VOC-exposed pathogen. GC-MS analysis revealed a complex volatile cocktail, including 1-pentanol, 1-propanol, ethyl hexanol, ethanol, 2-methyl-1-butanol, ethyl acetate, dimethyl trisulfide, p-xylene, styrene, and 1,4-pentadiene. Moreover, authors applied some of these synthetic compounds singularly, and achieved significant inhibition of *Aspergillus* growth and AFB1 production [96].

Finally, a recent study carried out by Yang et al. [99] has pointed out the biocontrol efficacy of VOCs released by an edible strain of *S. cerevisiae*, isolated from pomace of *Ficus carica*, on the growth and aflatoxin production of *A. flavus*. The VOCs-exposed mycelium morphology was distorted, and cell-membrane damage was observed. GC/MS analysis indicated that *S. cerevisiae* volatilome includes 9 compounds, with ethanol and 3-methyl-1-butanol accounting for 51.98% and 33.22%, respectively. In the monomer experiments, 3-methyl-1-butanol showed the highest inhibitory effect on *A. flavus* [99].

### 3.3. Fusarium

Species within this Genus produce three important classes of mycotoxins, i.e., trichothecenes, zearalenone (ZEN), and fumonisins (FBs). These mycotoxins can be found on a wide variety of agricultural commodities, but represent a major concern for the cereal sector, due to their widespread occurrence and to the difficulty in mitigating contamination in cereals and cereal-derived products (food, feed, beverages), since they accumulate in grains during the crop cycle and are only partially removed by processing [123]. Trichothecenes are grouped in two classes based on the presence (B-trichothecenes) or absence (A-trichothecenes) of a keto group at the C-8 position. Type A-trichothecenes include T-2 toxin (T-2), HT-2 toxin (HT-2), diacetoxyscirpenol (DAS), and neosolaniol (NEO). Among type B-trichothecenes, those having significant implications for health issues are: deoxynivalenol (DON), nivalenol (NIV), and their acetylated derivatives 3-acetyldeoxynivalenol (3-ADON), 15-acetyldeoxynivalenol (15-ADON), and 4-acetylnivalenol (4-ANIV, syn. fusarenone-X [124]). Trichothecenes may cause digestive disorders, reproductive toxicities, weight loss, decreased immunity, decreased plasma glucose levels, and pathological changes in the liver and stomach. Among the most relevant trichothecene-producing species are *F. graminearum*, *F. culmorum*, *F. crookwellense*, *F. equiseti*, *F. poae*, *F. langsethiae*, and *F. sporotrichioides*. ZEN is immunotoxic, hepatotoxic, hematotoxic, nephrotoxic, and has been associated with hepatocarcinoma. Moreover, ZEN has estrogenic effects, and ingestion of ZEN-contaminated feed induces damage in the reproductive tract, uterine enlargement, and reduced fertility [125]. Some of the above-mentioned DON-producing species, such as *F. graminearum*, *F. culmorum*, *F. equiseti*, and *F. crookwellense*, may also produce ZEN. FUM (see *Aspergillus*) are mainly produced by *F. verticillioides* and *F. proliferatum* [126]. Additionally, *Fusarium* species may produce the so-called “emerging mycotoxins” (e.g., enniatins, beauvericin, moniliformin, and fusaproliferin) that are neither routinely analysed nor legislatively regulated, but whose occurrence is being increasingly reported [127].

The inhibitory potential of a marine isolate of *Debaryomyces hansenii* was assessed for the first time on the growth of *F. proliferatum* and *F. subglutinans* on maize grains. VOCs released by this strain inhibited mycelial development of the two tested pathogens by 54.2 and 43.5%, respectively, compared with control [101].

Zeidan et al. [90] exposed a DON-producing isolate of *F. graminearum* to VOCs released by colonies of *L. thermotolerans* grown *in vitro*, achieving up to 46% inhibition of radial colony growth and up to 93% reduction of DON production (i.e., 18.29 μg/kg as compared with the unexposed control containing 254 μg/kg).

In the same previously reported paper [69], *in vitro* growth of *F. cerealis* and *F. poae* was inhibited up to 85% by volatiles produced by *I. orientalis*, *P. occidentalis*, *M. guilliermondii*, and *M. caribbica*.

Recently, Podgórska-Kryszczuk et al. [102] tested the VOCs released by a collection of 28 *M. guilliermondii*, *Cyberlindnera saturnus*, *Rhodotorula glutinis* and *Cryptococcus carnescens* representative isolates, including culture-collection strains and isolates from conventional and organic wheat, against vegetative growth and spore germination of *F. culmorum*, *F. graminearum* and *F. poae in vitro*. The tested strains exhibited variable biological control activity against the three *Fusarium* species, but VOCs emitted by an isolate of *C. carnescens* obtained from organic wheat seeds showed consistent inhibition of all three *Fusaria* [102].

### 3.4. Penicillium

This genus includes about 350 species occurring worldwide, some causing relevant damage to stored commodities, where they produce a wide range of mycotoxins. *Penicillium expansum*, incitant of the blue-mould decay in pome and stone fruits, is the most frequent pathogen isolated on rotten apples, pears, plums, peaches, apricots, cherries, blackcurrants, grapes, melons, and strawberries [128]. Many other species of *Penicillium* can be isolated from decayed fresh fruit and vegetables, e.g., *P. aurantiogriseum*, *P. brevicompactum*, *P. citrinum*, *P. crustosum*, *P. cyclopium*, *P.* *funiculosum*, *P. glabrum*, *P. griseofulvum*, *P. purpurogenum*, *P. olsonii*, *P. thomii*, *P. tularense*, and *P. viridicatum* [128]; whereas *P. carneum*, *P. nordicum*, *P. roqueforti*, and *P. verrucosum* commonly colonise stored cereals [129]. The most frequently reported *Penicillium* mycotoxin associated with fruit and vegetable decay is patulin (PAT), which represents the major mycotoxin produced by *P. expansum*, although it can also be produced by other species, such as *P. chrysogenum*, *P. cyclopium*, *P. cyaneo-fulvum*, and *P. griseofulvum*. PAT ingestion results in severe toxicosis, including mutagenic, teratogenic, hepatotoxic, nephrotoxic, neurotoxic and genotoxic effects. There is no experimental evidence that PAT has a carcinogenic effect on animal models, and therefore IARC classified it in Group 3 (“not carcinogenic to humans”). Yet the ability of PAT to cause gene mutations in mammalian cells has been reported [130], and this might lead to a revision of its IARC classification. Besides PAT, other mycotoxins are produced by *Penicillium* species during colonisation of stored commodities, these include, albeit not exclusively, citrinin, penicillic acid, cyclopiazonic acid and penitrem. Other *Penicillium* toxic secondary metabolites are chaetoglobosins, communesins, roquefortine C, and expansolides [131].

In a pioneering study, Björnberg and Schnürer [103] first evaluated the efficacy of living cells of *P. anomala* to control grain-storage moulds *in vitro* (*P. roqueforti* and *Aspergillus candidus*). While they did not specifically investigate the activity of yeast VOCs, they mentioned in the discussion that “Volatile metabolites could constitute another source of antagonism in this system. Our strain of *Pichia anomala* produces large amounts of ethyl acetate as do other *H. anomala* strains (syn. *Pichia anomala* [132]). This compound is known to reduce mold growth and spore germination [133]. The ability of even low numbers of yeast to alter the appearance of mold colonies might be due to the presence of inhibitors in the gas phase. Other volatile and nonvolatile inhibitory metabolites may also be produced”. To the best of our knowledge, this is the first mention of the presumed role of yeast VOCs in the control of postharvest pathogens.

More than 10 years later, Ädel Druvefors and Schnürer [104] and Ädel Druvefors et al. [105] confirmed the biocontrol capability of the same strain of *P. anomala*, along with representatives of 57 other yeast species against *P. roqueforti* in a semi-airtight grain mini-silo system. None of the tested strains showed a better efficacy compared with the original *P. anomala* strain J121, yet some new species (*Candida fennica*, *Candida pelliculosa*, *Candida silvicultrix*, *Pichia burtonii*, *Pichia farinose*, and *Pichia membranifaciens*) strongly inhibited the development of *P. roqueforti* in the mini silos [104]. The role of ethyl acetate was supposed to be relevant in the biocontrol efficacy of the tested yeasts. Moreover, Ädel Druvefors et al. [105] demonstrated the enhancement of the inhibitory efficacy of *P. anomala* during airtight storage of wheat when complex medium, maltose, or glucose were added. The products of glycolysis, mainly ethanol and ethyl acetate, were shown to increase upon addition of glucose to *P. anomala*-inoculated wheat grain, suggesting that the mould-inhibiting effect could be due to the antifungal action of these two metabolites. This result offers new insights on the formulation of biocontrol yeast, since sugar amendments may enhance their efficacy, particularly when volatiles derived from sugar metabolism are involved in the inhibition of storage pathogens in airtight systems.

Other yeast species (*Candida maltosa*, *D. hansenii*, *I. orientalis*, *P. occidentalis*, *M. guilliermondii*, *M. caribbica*, *K. marxianus*) have been later tested *in vitro* against grain-spoiling fungi such as *P. citrinum*, *P. chrysogenum*, *P. roqueforti*, and *P. verrucosum* [66,69,70,106,107]. In most instances, the biological efficacy of volatile compounds was measured on vegetative growth or spore germination (Table 1). Only Alasmar et al. [70] reported a significant reduction (from 31.1 ± 0.54 μg/kg in the control, to 0.12 ± 0.03 μg/kg in the exposed fungus) of OTA released in the medium by *P. verrucosum* upon exposure to *K. marxianus* volatiles. The volatile components considered responsible for fungal growth or mycotoxin inhibition were: isoamyl acetate, isoamyl alcohol [66], 2-methyl-1-propanol, 3-methyl-1-butanol, 2-methyl-1-butanol [106], 2-phenylethanol [69,107], phenylethyl acetate [69], nonadecane, eicosane, docosane, heptacosane, hexatriacontane, and tetracosane [70] acetone [107], as well as several esters, alcohols, terpenes, ketones, aldehydes, and aromatic hydrocarbons [69].

Due to its widespread presence as fruit decay incitant, and to the frequent contamination of fresh apple and pear fruit (and derived baby food and beverages) with PAT, *P. expansum* has emerged as a classical-model system in the biological control of postharvest pathogens [107,134]. Accordingly, several reports on the key role of yeast VOCs among the biocontrol mechanisms of action deal with this pathogen (Table 1).

Mari et al. [108] first reported the inhibitory efficacy (albeit low: 17.6–27.6%) of VOCs emitted by two strains of *A. pullulans* on the mycelium growth of *P. expansum*. The same strains were further characterised by the same research group to better ascertain their role in the yeast biocontrol properties towards *Penicillium* spp., including *P. expansum*; conidia germination was completely inhibited by VOCs produced by *A. pullulans*. Moreover, artificially inoculated apples were biofumigated with VOCs emitted by both antagonists and apple decay caused by *P. expansum* was significantly reduced (by 74%), confirming the results obtained *in vitro*. Yeast VOC composition was qualitatively evaluated by headspace (HS)-SPME GC-MS and revealed the presence of a complex blend of molecules, with 2-phenylethanol showing the highest relative peak area, followed by 3-methyl-1-butanol, 2-methyl-1-butanol, and 2-methyl-1-propanol alcohols [60]. Pure synthetic compounds were tested on spore germination and confirmed their inhibitory activity: 2-phenylethanol was the most active (EC_50_ value: 0.79 μL-1 headspace), followed by 2-methyl-1-butanol (0.89), 3-methyl-1-butanol (1.01), and 2-methyl-1-propanol (1.28).

A significant inhibitory activity (62–100%) towards *in vitro* radial colony growth of *P. expansum* was reported by Lutz et al. [109], who tested the biocontrol potential of two epiphytic yeasts (*Cryptococcus victoriae* and *Naganishia albida*—syn. *C. albidus*) isolated from pear fruit during cold postharvest storage in two packinghouses in North Patagonia, Argentina.

Agirman and Erten [110] evaluated the biocontrol activity of *A. pullulans* and *M. guillermondii* against *P. expansum*. Yeast VOCs inhibited the radial colony development of the pathogens by up to 56% at pH 4.5.

Finally, Gomomo et al. [111] screened 104 yeast isolates in search of potential antagonists against different postharvest pathogens of fruit, including *P. expansum*. The isolates had variable bioactivity towards the three pathogens, with three isolates (*Candida pyralidae*, *M. guilliermondii*, and *P. kluyveri*) displaying the highest biocontrol potential through the emission of VOCs, with percent inhibition ranging from 69 to 81% [111].

Surprisingly, none of the available reports on yeast VOC efficacy against *P. expansum* presents any evidence for the reduction of patulin production *in vitro* or *in vivo*. While the yeast-mediated conversion of patulin into the less-toxic metabolites desoxypatulinic acid and ascladiol was demonstrated [134,135,136,137,138], it would be interesting to evaluate whether yeast VOCs may downregulate genes involved in patulin biosynthesis in *P. expansum*, as it has been demonstrated by Wang et al. [139] upon exposure to the essential oils cinnamaldehyde and citral.

This aspect is worthy of clarification, since Pennerman et al. [140] have reported that *P. expansum* exposed to sub-inhibitory levels of the fungal volatile 1-octen-3-ol (a germination and growth inhibitor in several species) actually increases the biosynthesis of patulin in a medium that normally suppresses the mycotoxin. Similarly, Galván et al. [97] pointed out that low concentrations of 2-phenylethyl acetate and furfuryl acetate determined an increase in AFs and OTA synthesis by *A. flavus* and *A. niger*, respectively.

## 4. Conclusions and Perspectives

Postharvest applications of microbial volatilome are being increasingly explored, as air-tight conditions that can be more easily achieved during storage allow the homogeneous saturation of the atmosphere (at the silo, container, bag, or package level) by the microbial volatiles well above the minimal effective concentration required to exert their fungistatic/fungitoxic effects, even in cases of poor accessibility of the target fungus [52]. Yeast VOCs are generally non-toxic, and mostly recognised as GRAS (Generally Regarded As Safe); the integration of these volatile compounds into the postharvest chain would not incur problems related to residues or environmental accumulation—which are typical drawbacks in the application of synthetic fungicides—thereby allowing effective, safe protection of agricultural commodities [51].

VOC-producing yeasts may be used directly as BCAs, by treating fruit with living cells, or by including cultures into active packaging, shipping boxes, or containers [27]. Recently, an apple-based edible coating has been proposed to incorporate antagonistic *M. pulcherrima*: an apple-pomace-residue film was applied as a bioactive coating on apple fruits inoculated with *P. expansum*, significantly reducing both pathogen growth and patulin production during storage [141]. Alternatively, bioactive volatiles or volatile-producing yeasts may be formulated by encapsulation methods, allowing both triggered and controlled release [142]. Parafati et al. [143] immobilised VOC-emitting *W. anomalus*, *M. pulcherrima*, *A. pullulans* and *S. cerevisiae* on commercial hydrogel spheres. Experimental trials were performed on strawberry and mandarin fruits inoculated with *B. cinerea* and *P. digitatum*, respectively, and proved the ability of VOCs to significantly reduce postharvest decay on artificially wounded tissues. Although these experiments did not involve mycotoxigenic fungi, it is reasonable to hypothesise that hydrogel spheres used as a support for VOC-emitting yeasts may open new perspectives to the employment of this polymer as a biofumigant in postharvest packaging to control mycotoxin contaminants. Also, incorporating VOCs in specialised slow-release materials such as polyethylene terephthalate, and packing in sachets or pouches to be placed into food storage boxes or bags represents an effective alternative to control spoilage during the complete commercialisation and storage chain [144]. All these applications present a great deal of challenges, due to the intrinsic volatility of the active ingredients, but at the same time they offer the possibility of developing brand new basic knowledge, experimental evidence, and technical solutions to achieve maximum (bio)control efficacy at an affordable price. The question arises as to whether the application of synthetic VOCs—albeit having a natural equivalent—could still be considered as “biological control”. These speculations, though, are beyond the objective of the present review.

Many studies highlighted the need to analyse VOCs production by a given microorganism under different conditions (different nutrient media, different growing times, different cell concentrations, presence vs. absence of the target pathogen), since environmental stimuli may exert a great influence on the specific cocktail of exometabolites, including volatile ones [68,145,146]. Consistency of the VOCs’ inhibitory efficacy appears particularly relevant in areas of the world that are affected by climate change, since mycotoxigenic fungi may be stimulated to produce increased mycotoxin levels in response to climate-related abiotic factors [147,148].

The genetic improvement of VOC-producing strains may help stabilise the volatile emissions, thereby improving their biocontrol potential regardless of the climatic or storage conditions. Mutant strains with increased production of VOCs could be generated from selected BCAs; recently, a promising approach has been reported on the yeast *Saprochaete suaveolens* (syn. *Geotrichum fragrans*), able to produce α-unsaturated esters from branched-chain amino acids (BCAAs: e.g., isoleucine, leucine and valine), such as isobutyl, isoamyl, or ethyl tiglate, which are rarely found in other yeasts. Since β-oxidation allows the growth of this strain on BCAAs as the sole carbon source, mutants were generated that could no longer grow on BCAAs, while redirecting the carbon flow towards esterification of α-unsaturated esters. Among a collection of 15,000 UV-irradiated clones, 9 mutants were unable to grow on BCAAs and one was able to produce eight times more VOCs as compared with the wild-type [149]. This approach may offer the opportunity to improve the efficacy of existing biocontrol yeasts whose mechanism of action is based on the production of VOCs by generating UV mutants that are not concerned by the regulation applicable for GMOs, due to their long-standing safety record.

In conclusion, research on the role of yeast VOCs in the biocontrol of mycotoxigenic postharvest fungi has greatly progressed during recent years. The increasing range of new, effective yeast strains whose mechanism of action includes, or is based on, the emission of bioactive volatile compounds, along with the development of sensitive metabolomics approaches [150,151,152], will expand the arsenal of new molecules showing great potential for practical application in the control of mycotoxin-producing spoilage fungi. Despite our growing knowledge on the structure–activity relationship of yeast volatilome, further studies are still needed to better define the food safety risks associated with VOCs affecting multiple metabolic processes in the target fungi. Moreover, we ought to address our scant information on the mechanisms underlying the regulation and expression of the fungal secondary metabolome. Considering the crucial role of volatiles in food and perfume industry, this area appears to be a bountiful source of hints to expand the knowledge of future generations of scientists.

## Figures and Tables

**Table 1:** Synopsis of the most relevant literature reporting on volatile-emitting yeast antagonists applied to control mycotoxigenic fungi.

Target Fungus	Mycotoxin	Major Volatile(s)	Emitting Yeast	Mechanism/Function Inhibited	Commodity or Experimental Setup	Reference
*Alternaria alternata*, *A. arborescens*, *A. tenuissima*	N.D.	3-methylbutyl hexanoate, 3-methylbutylpentanoate, 2-methylpropyl hexanoate, pentylhexanoate	*Candida sake*	Vegetative growth, infection and fruit rot	*In vitro* and apple fruit	[72]
*Alternaria alternata*	N.D.	ethyl acetate, isoamyl acetate, ethyl butyrate, ethyl hexanoate, phenylethyl acetate, 2-phenylethanol, isobutanol	*Metschnikowia pulcherrima*, *Saccharomyces cerevisiae*, *Wickerhamomyces anomalus* (syn. *Pichia anomala*)	Vegetative growth, infection and fruit rot	*In vitro* and strawberry fruit	[67]
*Alternaria alternata*	N.D.	ethanol, 2-methyl-1-propanol, 3-methyl-1-butanol, 2-phenylethanol	*Aureobasidium pullulans*	Spore germination, vegetative growth, membrane permeability, cell wall integrity	*In vitro*	[63,78]
*Aspergillus ochraceus*	OTA	ethyl acetate, isobutyl acetate, 2-phenyl ethyl acetate, ethyl propionate, isoamyl alcohol	*Pichia anomala*, *Pichia kluyveri*, *Hanseniaspora uvarum*	Spore germination, vegetative growth, OTA biosynthesis	*In vitro*	[64,65]
*Aspergillus carbonarius*	OTA	N.D.	*Candida friedrichii*, *Candida intermedia*, *Cyberlindnera jadinii*, *Lachancea thermotolerans*	Vegetative growth, sporulation, infection, OTA biosynthesis	*In vitro* and grape berries	[87]
*Aspergillus carbonarius*, *A. caelatus* *A. terreus*, *A. versicolor*	N.D.	N.D.	*Saccharomyces cerevisiae*, *S. kluyveri*, *Candida sake*, *Schwanniomyces vanrijiae*, *Wickerhamiella versatilis* (Syn. *Candida versatilis*),	Vegetative growth	*In vitro*	[88]
*Aspergillus carbonarius*, *A. ochraceus*	OTA	2-phenylethanol	*Candida friedrichii*, *Candida intermedia*, *Cyberlindnera jadinii*, *Lachancea thermotolerans*	Vegetative growth, sporulation, gene expression, OTA biosynthesis	*In vitro*, *in silico*	[61]
*Aspergillus carbonarius*	OTA	2-phenylethanol	*Candida intermedia*	Vegetative growth, OTA biosynthesis, fungal metabolism	*In vitro*, *in silico*, proteome analysis	[62]
*Aspergillus carbonarius*	OTA	nonadecane, eicosane, docosane, heptacosane, hexatriacontane, and tetracosane	*Kluyveromyces marxianus*	OTA biosynthesis, infection	*In vitro* and grape berries	[70]
*Aspergillus carbonarius*, *A. ochraceus*	OTA	2-methyl-1-butanol, 3-methyl-1-butanol, 3-methylbutyl acetate, ethyl octanoate, 2-nonanone	*Saccharomyces cerevisiae*	OTA biosynthesis, infection	*In vitro* and coffee beans	[89]
*Aspergillus flavus*	AFs	2-phenylethanol	*Pichia anomala*	Spore germination, vegetative growth, AF biosynthesis, gene expression	*In vitro*, *in silico*	[57]
*Aspergillus flavus*	N.D.	2-phenylethanol	*Wickerhamomyces anomalus* (syn. *Pichia anomala*)	Vegetative growth, AF biosynthesis, gene expression	*In vitro*, *in silico*	[59]
*Aspergillus parasiticus*	AFs	2-phenylethanol	*Lachancea thermotolerans*	Vegetative growth, AF biosynthesis	*In vitro*	[90]
*Aspergillus flavus*	N.D.	2-phenylethanol, phenylethyl acetate, several esters, alcohols, terpenes, ketones, aldehydes, and aromatic hydrocarbons	*Issatchenkia orientalis* (syn. *Pichia kudriavzevii*), *Pichia occidentalis*, *Meyerozyma guilliermondii*, *Meyerozyma caribbica*	Vegetative growth	*In vitro*	[69]
*Aspergillus flavus*	AFB1	N.D.	*Issatchenkia orientalis* (syn. *Pichia kudriavzevii*), *Lachancea thermotolerans*	Vegetative growth, AF biosynthesis	*In vitro*	[91]
*Aspergillus flavus*	AFB1	1-pentanol	*Candida nivariensis*	Spore germination, vegetative growth, AF biosynthesis	*In vitro*	[92]
*Aspergillus flavus*	AFB1	3-methyl-1-butanol, 2-methyl-1-butanol, hydrazine-1-1-dimethyl, butanoic acid-3-methyl	*Kwoniella heveanensis*	Vegetative growth, sporulation, AF biosynthesis	*In vitro* and maize kernels	[93]
*Aspergillus flavus*	AFs	N.D.	*Candida friedrichii*, *Candida intermedia*, *Cyberlindnera jadinii*, *Lachancea thermotolerans*	Vegetative growth, AF biosynthesis, gene expression	*In vitro*, *in silico*	[94]
*Aspergillus flavus*	AFB1, AFB2	acetic acid, 2-methylbutanoic acid, isobutyric acid, 2-methylbutanol, isoamyl alcohol, 2-methyl-1-butanol, 2-phenylethanol, ethyl acetate, isoamyl acetate, 2-phenylethyl acetate, 2-methylbutyl acetate	*Hanseniaspora opuntiae*, *Hanseniaspora uvarum*	Vegetative growth, AF biosynthesis, gene expression	*In vitro*, *in silico*	[95]
*Aspergillus flavus*	AFB1	1-pentanol, 1-propanol, ethyl hexanol, ethanol, 2-methyl-1-butanol, ethyl acetate, dimethyl trisulfide, p-xylene, styrene, 1,4-pentadiene, ethyl acetate, hexanal, 1-propanol, 1-heptanol, 1-butanol, benzothiazole	*Candida tropicalis* *Issatchenkia orientalis* (syn. *Pichia kudriavzevii*), *Saccharomyces cerevisiae*, *Suhomyces xylopsoci*	Vegetative growth, AF biosynthesis	*In vitro*	[96]
*Aspergillus flavus*, *A. niger*	OTA, AFs	furfuryl acetate, 2-phenylethyl acetate	*Hanseniaspora opuntiae*, *Hanseniaspora uvarum*	Spore germination, vegetative growth, infection, OTA and AF biosynthesis, gene expression	*In vitro*, *in silico*, and dried figs	[97]
*Aspergillus flavus*	N.D.	N.D.	*Issatchenkia orientalis* (syn. *Pichia kudriavzevii*), *Saccharomyces cerevisiae*	Spore germination, vegetative growth	*In vitro*	[98]
*Aspergillus flavus*	AF	acetaldehyde, 2 ethyl acetate, ethanol, 1-propanol, 2-methyl-1-propanol, 3-methyl-1-butanol, 2-pentanol, acetic acid, 2-phenyl ethanol	*Saccharomyces cerevisiae*	Spore germination, vegetative growth, sporulation, infection cell membrane permeability, cell wall integrity, AF biosynthesis	*In vitro* and walnut	[99]
*Aspergillus parasiticus*, *A. niger*, *A. verrucosum*	N.D.	2-phenylethanol	*Cyberlindnera jadinii*	Vegetative growth	*In vitro*	[100]
*Fusarium proliferatum*, *F. subglutinans*	N.D.	N.D.	*Debaryomyces hansenii*	Vegetative growth	*In vitro*	[101]
*Fusarium graminearum*	DON	2-phenylethanol	*Lachancea thermotolerans*	Vegetative growth, DON biosynthesis	*In vitro*	[90]
*Fusarium cerealis*, *F. poae*	N.D.	2-phenylethanol, phenylethyl acetate, several esters, alcohols, terpenes, ketones, aldehydes, and aromatic hydrocarbons	*Issatchenkia orientalis* (syn. *Pichia kudriavzevii*), *Pichia occidentalis*, *Meyerozyma guilliermondii*, *Meyerozyma caribbica*	Vegetative growth	*In vitro*	[69]
*Fusarium culmorum*, *F. graminearum*, *F. poae*	N.D.	N.D.	*Meyerozyma guilliermondii Cyberlindnera saturnus*, *Rhodotorula glutinis*, *Cryptococcus carnescens*	Vegetative growth	*In vitro*	[102]
*Penicillium roqueforti*, *Aspergillus candidus*		ethyl acetate	*Pichia anomala*	Vegetative growth	*In vitro*	[103]
*Penicillium roqueforti*	N.D.	ethanol, ethyl acetate	*Pichia anomala*	Infection	Wheat grain	[104,105]
*Penicillium citrinum*, *Penicillium chrysogenum*	N.D.	isoamyl acetate, isoamyl alcohol	*Candida maltosa*	Spore germination	*In vitro*	[66]
*Penicillium verrucosum*	N.D.	2-methyl-1-propanol, 3-methyl-1-butanol and 2-methyl-1-butanol	*Debaryomyces hansenii*	Vegetative growth	*In vitro*	[106]
*Penicillium* *chrysogenum*, *Penicillium expansum*	N.D.	2-phenylethanol, phenylethyl acetate, several esters, alcohols, terpenes, ketones, aldehydes, and aromatic hydrocarbons	*Issatchenkia orientalis* (syn. *Pichia kudriavzevii*), *Pichia occidentalis*, *Meyerozyma guilliermondii*, *Meyerozyma caribbica*	Vegetative growth	*In vitro*	[69]
*Penicillium verrucosum*	OTA	nonadecane, eicosane, docosane, heptacosane, hexatriacontane, and tetracosane	*Kluyveromyces marxianus*	OTA biosynthesis, vegetative growth	*In vitro*	[70]
*Penicillium roqueforti*	N.D.	2-phenylethanol, acetone	*Debaryomyces hansenii*	Spore germination, vegetative growth	*In vitro*	[107]
*Penicillium expansum*	N.D.	N.D.	*Aureobasidium pullulans*	Vegetative growth	*In vitro*	[108]
*Penicillium expansum*	N.D.	N.D.	*Cryptococcus victoriae*, *Naganishia albida* (syn. *C. albidus*)	Spore germination, vegetative growth, infection	*In vitro* and pear fruit	[109]
*Penicillium expansum*	N.D.	3-methyl-1-butanol, 2-methyl-1-butanol, 2-methyl-1-propanol, 2-phenylethanol	*Aureobasidium pullulans*	Spore germination, vegetative growth, infection	*In vitro* and apple fruit	[60]
*Penicillium expansum*	N.D.	N.D.	*Aureobasidium pullulans; Meyerozyma guilliermondii*	Vegetative growth	*In vitro*	[110]
*Penicillium expansum*	N.D.	N.D.	*Candida pyralidae*, *Meyerozyma guilliermondii*, *Pichia kluyveri*	Vegetative growth	*In vitro*	[111]

N.D.: not determined; OTA: ochratoxin A; AF: aflatoxin; DON: deoxynivalenol.
